# Translational Dynamics and Structural Enhancement Effect in High-Temperature Supramolecular Systems of Asparaginyl Low-Molecular-Weight Gelators and Propylene Carbonate

**DOI:** 10.1021/acs.macromol.4c03225

**Published:** 2025-09-05

**Authors:** Farooq Ahmad, Natalia Bielejewska, Dawid Pakulski, Michał Bielejewski

**Affiliations:** † Institute of Molecular Physics, 119441 Polish Academy of Sciences, M. Smoluchowskiego 17, Poznań 60-179, Poland; ‡ Centre for Advanced Technologies, Adam Mickiewicz University, Uniwersytetu Poznańskiego 10, Poznań 61-614, Poland

## Abstract

Chemical engineering paves the way for the design of new materials with targeted properties. Supramolecular chemistry allows the creation of molecular assemblies from small molecules based on noncovalent interactions between the components, e.g., hydrogen bonds and dispersive or electrostatic forces, leading to the templating of self-assembly structures. The reversible bonding interaction allows for optimization of the final structure and enhancement of the properties. In this study, we have used this approach to design and prepare supramolecular membranes that work at temperatures exceeding the 100 °C limit. The system is based on low molecular weight gelators (LMWGs), which provide versatility of compositions, flexibility, tunable properties, and improved sustainability. LMWG-based systems mostly exhibit gel-like features, offering an alternative with enhanced responsiveness, self-healing abilities, recyclability, and viscoelastic properties. In this context, we developed a gel-like membrane using an aspartame derivative as the LMWG and propylene carbonate as a liquid phase to prepare systems that can solidify carbonate solvents widely used in the chemical industry. The TGA/DTG and DSC thermal analyses were used to evaluate the system’s performance at high temperatures. The intermolecular interactions, gelation mechanism, and solvent dynamics were examined using different nuclear magnetic resonance methods. The microstructures of the obtained membranes were studied by using a fluorescence confocal scanning microscope. The obtained results have shown that the designed systems subjected to proper thermal processing routes can achieve enhanced structural stability, allowing them to work continuously at temperatures exceeding the 100 °C limit. The NMR studies showed that translational dynamics of the solidified liquid remain comparable to those observed for the liquid state, preventing the bulk flow simultaneously and compensating for the thermal convection effects. The performed matrix analysis showed how the self-assembled supramolecular aggregates of the gelator evolve with the membrane composition. We have demonstrated that the investigated membranes exhibit efficient self-healing effects above some concentration threshold.

## Graphical abstract

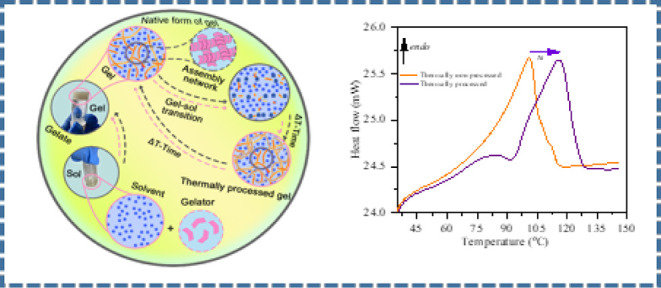

## Introduction

The state of matter in which materials exist under given external conditions often brings limitations in many application areas. Therefore, scientists and engineers have put much effort into developing new materials with targeted properties. In many cases, the most beneficial approach combines the advantages of liquid- and solid-state properties of matter while eliminating their disadvantages. The process of solidifying materials in the liquid state by, e.g., adding fillers, using polymerization reactions, embedding in porous materials, or using adsorbents, has contributed to increasing the safety level, mechanical resistance, and thermal stability of common substances used as liquids. It has also facilitated the development of the purification processes and improved waste management process.^1^ Simultaneously, the conversion from the liquid to solid state negatively influences transport properties, e.g., conductivity, reaction kinetics, recycling process, and recovery of the components. One way to overcome the common drawback of many solidification processes based on chemical bonding is to use physical interactions to immobilize molecules in supramolecular structures. The fast development of material engineering using supramolecular chemistry has contributed to designing advanced materials like covalent and metal–organic frameworks, carbon nanotube structures, nanomaterials with incorporated functional groups, etc.^2^ Shifting attention to organizing monomeric species into desirable superstructures made supramolecular chemistry a powerful tool for designing systems of self-assembled small molecules, creating three-dimensional superstructures.^3^ These structures that can immobilize many solvent molecules are called gels. To distinguish them from well-known polymer gels created upon chemical cross-linking reactions, irreversible in nature, they are called physical gels or molecular gels. Because gels, in general, may serve various purposes on both the microscopic and macroscopic levels (for example, in foods, fragrances, cosmetics, sports shoes, electrochemical devices, and chromatography), there has been much research toward creating more effective and adaptable small molecule gelators for use in the industry.^4^ Recently, molecular gels produced by low molecular weight gelators (LMWGs) have experienced high-speed development due to the increasing demand for materials with targeted properties for various applications and low influence on the natural environment. These can be set as hydrogels in aquatic media, organic gels in organic media, or organic and water-based environments.^5^

The gelation mechanisms for LMWG-based systems are driven solely by noncovalent interactions such as hydrogen bonding, aromatic (π–π) interactions, van der Waals interactions, ionic or organometallic coordination interactions, or a combination of the entities mentioned above.^6,7^ The process relies on the continuous breakdown and assembly of aggregates created by these gelator molecules upon heating and cooling their mixtures with solvent molecules. This has corresponded to developing self-assembled fibrillary complexes, creating entangled three-dimensional networks with different topographies and strengths.^8,9^

The literature on the topic of designing and synthesizing low molecular weight gelators shows that it is exceedingly difficult to anticipate, based on the molecular structure, the capacity of molecules to gel a particular class of solvents (organic/aqueous) and the stability of those that self-assemble. Consequently, understanding molecular stuffing, a supramolecular assemblage of molecular aggregates, and a 3D macroscopic linkage prominent to gelation performance are some of the most pressing issues in the design, manufacturing, and application of LMWGs.^10–12^ It is believed that understanding and controlling the interactions at the surface of the gel matrix and the solvent molecules make it possible to influence the properties of the solvent and solute molecules in multicomponent gel systems, which can lead to gaining functionality and increasing the performance of solidified liquid systems.^13^ The size, shape, and topology of geometrical restrictions and the liquid-matrix interactions near the interior surfaces all impact the behavior of the confined liquids and species dissolved in them. This is why the liquid phase embedded in supramolecular matrices, often called simply a gel state, exhibits specific behavior and properties that deviate from the bulk state^14,15^ or prevent gelation. At the macroscopic level, the gel state influences the translational motion of liquid molecules most significantly, holding them back from bulk flow. At the microscopic level, the intermolecular interactions of the solvent and solute with the surface of the matrix cause orientational ordering of the molecules close to the surface, change the translational motion from 3D bulk diffusion to 2D surface diffusion, alter the interactions between the solvent and solute molecules, influence the LMWG molecules’ self-assembly process, and determine the supramolecular structure.^16–19^ Generally, many physical properties of the investigated material change upon conversion from the liquid to solid state. Some of the changes can be advantageous for applicational reasons, such as higher mechanical resistance, higher safety, no danger of leakage, and ease of processing and shaping of the product. On the other hand, others can be disadvantageous, such as decreased transport properties, difficulties in contact at the interfaces, inhomogeneous structures, and defects. As LMWGs are also known for their very low critical minimum concentration (CMC), allowing the formation of gels with gelator weight concentration even below 1 wt %, they often precipitate at higher concentrations. Moreover, the addition of dissociated ions significantly disturbs the gelation process, weakening the structure or preventing the system from gelation. Consequently, finding a suitable LMWG that can immobilize highly concentrated electrolytes and retain the gel phase at high temperatures is complex and rare.^20–23^

In this work, we have shown for the first time that the l-β-2-ethylhexylasparaginyl-l-phenylalanine low molecular weight gelator can be used to create supramolecular 3D structures upon a physical self-assembly process carried out in propylene carbonate, used as the solvent. The unique feature of the studied molecular system is its ability to preserve the gel-like phase at a very high weight concentration of the LMWG without precipitating, allowing for achieving high thermal and time stability at temperatures above 100 °C. Moreover, we have found that the initial thermal properties can be further improved by simple thermal processing, allowing us to reach a temperature of gel-to-sol phase transition as high as 120 °C for a 20 wt % LMWG component. This unique feature has been observed for the first time, and it is of great importance, as it allows us to think of utilizing this system in new thermally reversible solid electrolytes based on lithium ions able to work at high temperatures. The possibility of creating a supramolecular system able to serve as a renewable electrolyte based on lithium would be a big step forward in the reasonable and sustainable management of lithium resources. This work provides a reference system for developing a supramolecular gel electrolyte system. PC was deliberately chosen due to its widespread use in battery technology, making it an ideal candidate for designing and preparing new renewable high-temperature solid electrolytes in the form of supramolecular gel membranes for utilization in energy storage applications. Moreover, the presented studies of molecular dynamics, kinetics, and intramolecular interactions of embedded PC molecules indicate very high mobility in the supramolecular phase, resulting in potentially very high conductivity of the prepared electrolyte systems.

## Results and Discussion

The chemical structures of the gelator G2 and the solvent, propylene carbonate (PC), are presented in Figure 1. The PC solvent was chosen to represent technological materials essential in the battery industry. Its high molecular dipole moment of 4.9D, polar and aprotic nature, and high dielectric constant make it a suitable solvent for various salts. The high polarity of PC allows the creation of an effective solvation shell around metal cations, thereby creating an electrolyte solution. Due to this, it is not easy to find suitable LMWG molecules that can self-assemble in such an environment and create stable gel phases. As stability and durability are priorities in finding new potential materials for creating solid electrolytes, we have investigated the transport properties, structure, and decomposition processes in the obtained supramolecular systems to determine the safe working temperature range.

**Figure 1:**
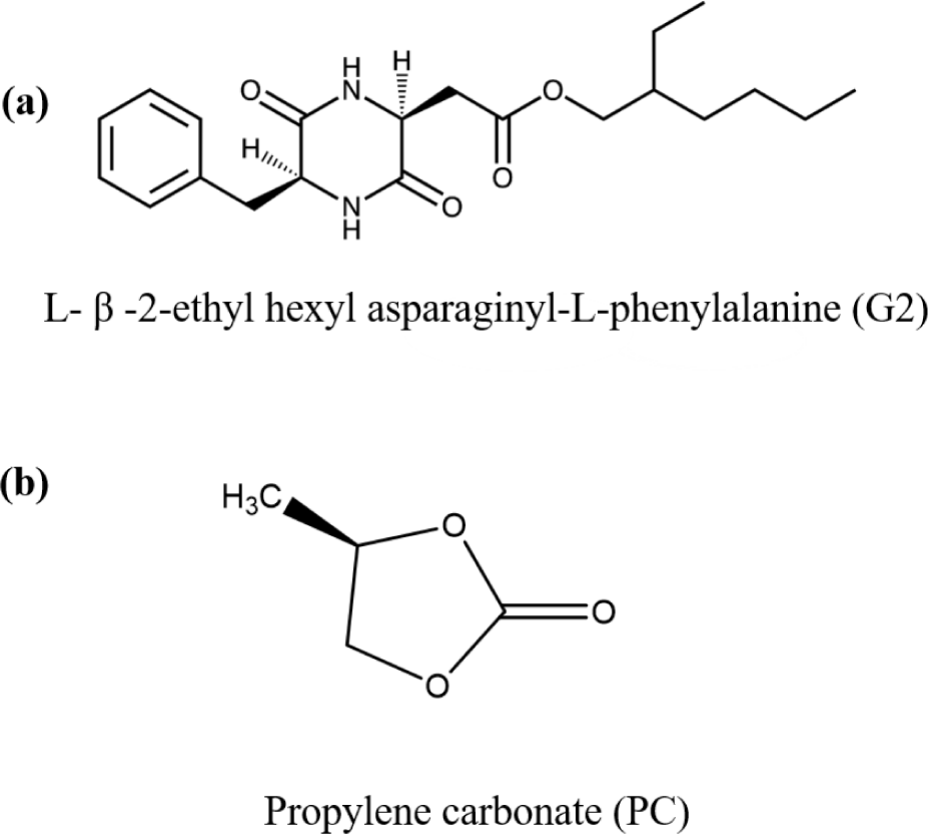
(a) The chemical structure of the LMWG molecule l-β-2-ethylhexyl asparaginyl-l-phenylalanine (G2) and (b) liquid propylene carbonate (PC).

### TGA/DSC

The thermal stability and decomposition processes for pure components and the obtained gel membranes were investigated and analyzed using TGA/DTG methods. Figure 2 shows recorded thermograms for the G2 gelator in solid state and gel phase at 20 wt % along with liquid PC.

**Figure 2:**
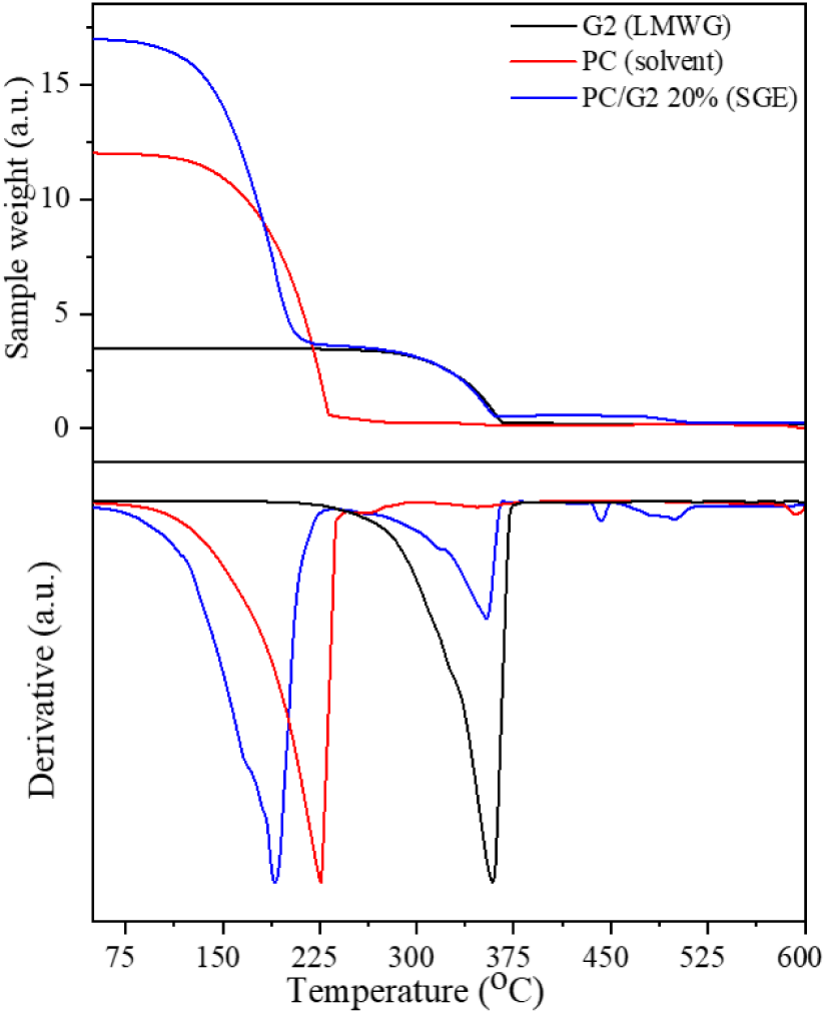
TGA thermograms (upper panel) and their derivative DTG curves (lower panel) for pure G2, solvent PC, and 20 wt % gel membrane measured at 10 K/min heating rate.

The DTG analysis indicated the onset of the G2 decomposition process at 220 °C with maximum kinetics at 360 °C. The evaporation of pure PC starts at 85 °C and achieves a maximum of 225 °C. In the 20 wt % gel membrane, the evaporation process of the solvent phase (PC) is shifted toward lower temperatures by 37 °C and shows a maximum at 188 °C. In contrast, the decomposition of G2 is observed at the same temperatures as in the solid-state case. Moreover, the DTG signal of PC evaporation in the gel sample shows observable step-like behavior, indicating that PC molecules are involved in intermolecular interactions of different strengths. In its neat form, propylene carbonate creates a network of hydrogen bonds between its molecules in the C–H···O=H configuration, forming head-to-tail correlated chains of molecules.^24^ In the gel membrane, these interactions are disturbed by the presence of the gelator matrix and interactions on its surface. As a result, the PC molecules near the surface of the gel matrix can form H-bonds with it, giving rise to shortening of the correlated chains of PC molecules. Therefore, the temperature of the evaporation process decreases in the gel phase, and the DTG curve displays observable steps in that dependence. The analysis of G2 in the PC thermogram shows no change in its decomposition temperature, indicating that the PC and possible intermolecular interactions do not influence the structure of G2. The DSC measurements were performed as a function of the G2 weight concentrations in PC to investigate the phase transition behavior in the obtained gel membranes. Figure 3 shows DSC curves for G2 and G2/PC at 20 wt % for direct investigation of the influence of interaction between PC and G2 on the observed phase transitions.

**Figure 3:**
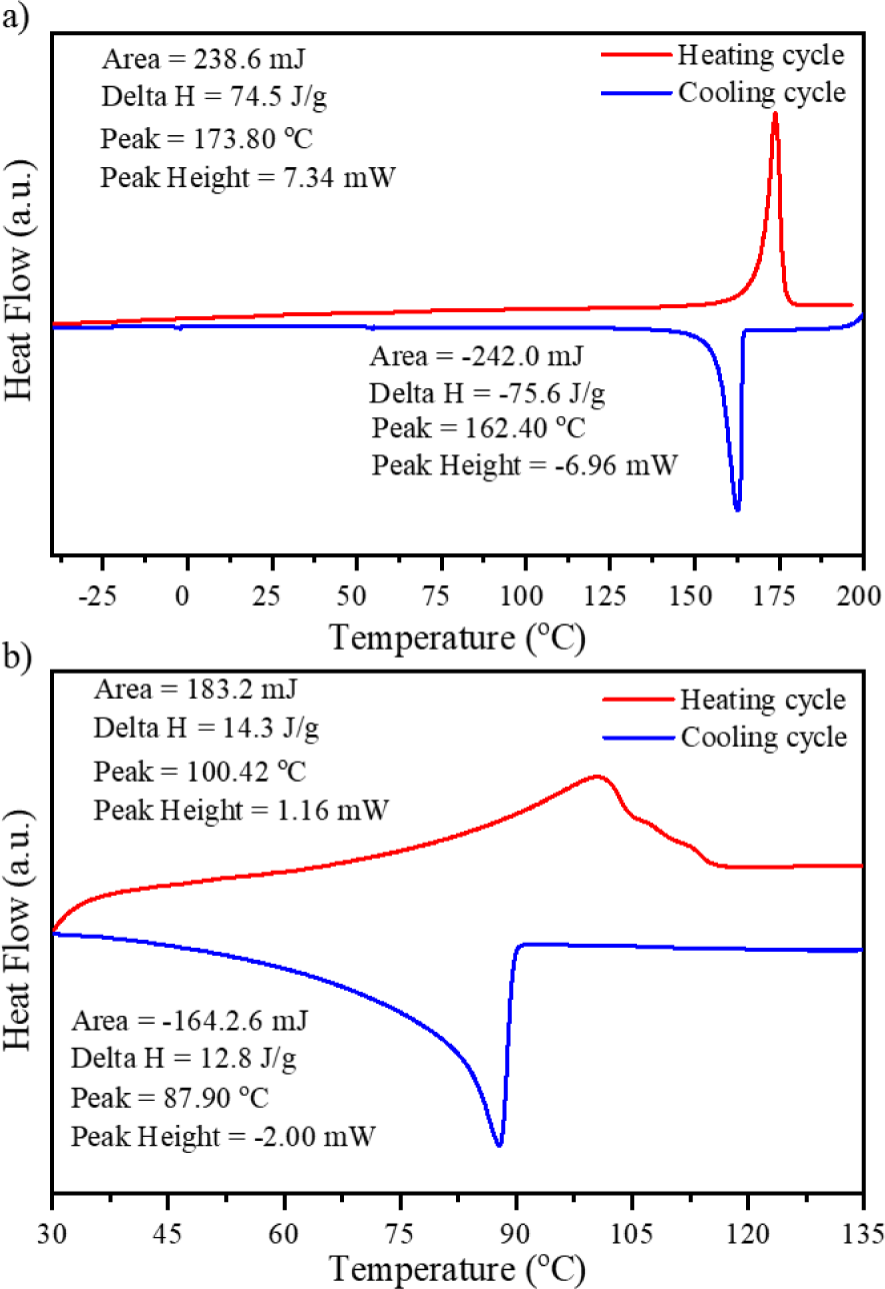
DSC curves for G2 (a) and G2/PC gel membrane at 20% weight concentration (b) measured at 10°/min heating/cooling temperature change rate.

In the case of the solid-state sample of G2, a sharp endothermic peak at 174 °C and a sharp exothermic peak at 162 °C in heating and cooling cycles were observed, respectively. The endothermic transition corresponds to the melting of G2, and the exothermic peak corresponds to crystallization. The corresponding phase transitions shift toward lower temperatures for the gel membrane sample. The endothermic phase transition is now related to melting the gel matrix composed of G2 molecules and shows a maximum of 100 °C. The peak becomes much broader with three distinguishable components at 106 and 112 °C. The enthalpy related to this process is also much lower than for solid-state G2. An exothermic peak is observed during cooling, with a maximum temperature of 88 °C. The peak is wider than solid-state G2 but shows no distinguishable components during the heating stage. The exothermic peak in the gel membrane is related to the gelation process originating from the self-assembly of the G2 molecules dispersed in PC. The enthalpy of this process is also much lower than that of solid-state G2. The decreased enthalpy of phase transitions in the gel membrane reflects the weaker interactions and bonding between G2 molecules, as the distances between individual molecules are much longer than in the solid state. To investigate the influence of the relative distance between gelator molecules on the endothermic and exothermic phase transitions, we have studied a series of gel membrane samples with different G2 molecule contents, resulting in weight concentrations of 1–50 wt %. The DSC curves for the heating and cooling stages in the gel membrane series are presented in Figure 4.

**Figure 4:**
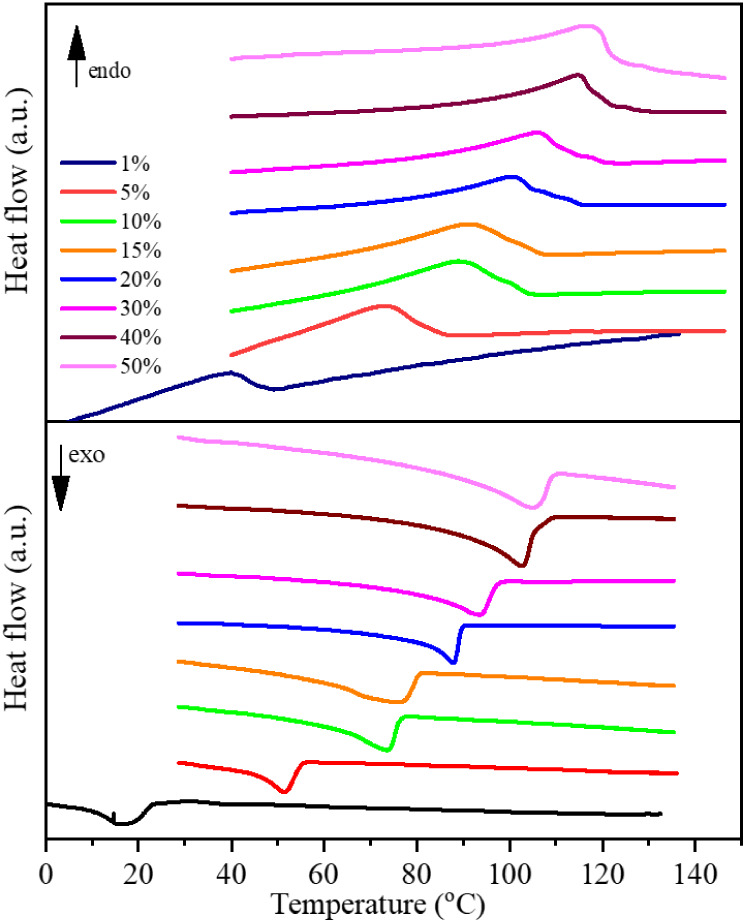
DSC curves during the heating (upper panel) and cooling (lower panel) states for the gel membrane series (1–50 wt % of G2).

The increased concentration of G2 molecules in prepared gel membranes increased the gel-to-sol phase transition temperature, which was reflected as an endothermic peak in the DSC heating curves. Moreover, for higher concentrations, the shape of that peak also changes, so distinguishable components observed for lower concentrations overlap. Also, the exothermic peak observed during cooling shifts significantly decreased the G2 concentration. To investigate the self-assembly process of G2 molecules in PC, we have calculated the enthalpy of the reversible gel-to-sol and sol-to-gel phase transitions using the Schroeder van Laar relation.^25^

1 $$\log\left(C_{m}\right)=-\frac{\Delta H}{2.303\cdot RT_{gs}}+const$$

where *C*_m_ is the molar gel concentration, *ΔH* is the melting enthalpy, *R* is the gas constant, and *T*_gs_ is the gel-to-sol phase transition temperature. For the first time, we also used this approach to describe the self-assembly of G2 molecules. The gelation process is related to creating physical intermolecular bonding between individual G2 molecules. Such bonds are continuously created and annihilated, depending on the system’s internal energy; either the creation or annihilation processes prevail. The gelation occurs when the creation of intermolecular bonds dominates. Moreover, the systems’ lower internal energy increases the bonds’ stability and durability, forming a rigid gel matrix. The determined enthalpy of the gelation process indicates the system’s highest internal energy, in which the creation of intermolecular bonds between G2 molecules prevails and triggers the gelation process. Figure 5 presents the temperature dependence of the phase transition temperatures for different molar concentrations of the studied gel membranes. The solid lines represent the best fit of eq 1 to the experimental data. The results from detailed DSC analysis showed that the enthalpies of self-disassembly and self-assembly are similar for gel membranes with G2 concentration above 10 wt %. This fact allows the gel membrane to effectively self-heal in case of partial melting upon local overheating. In the case of gel membranes with G2 concentration below 10%, the system’s temperature needs to be strongly reduced to self-heal.

**Figure 5:**
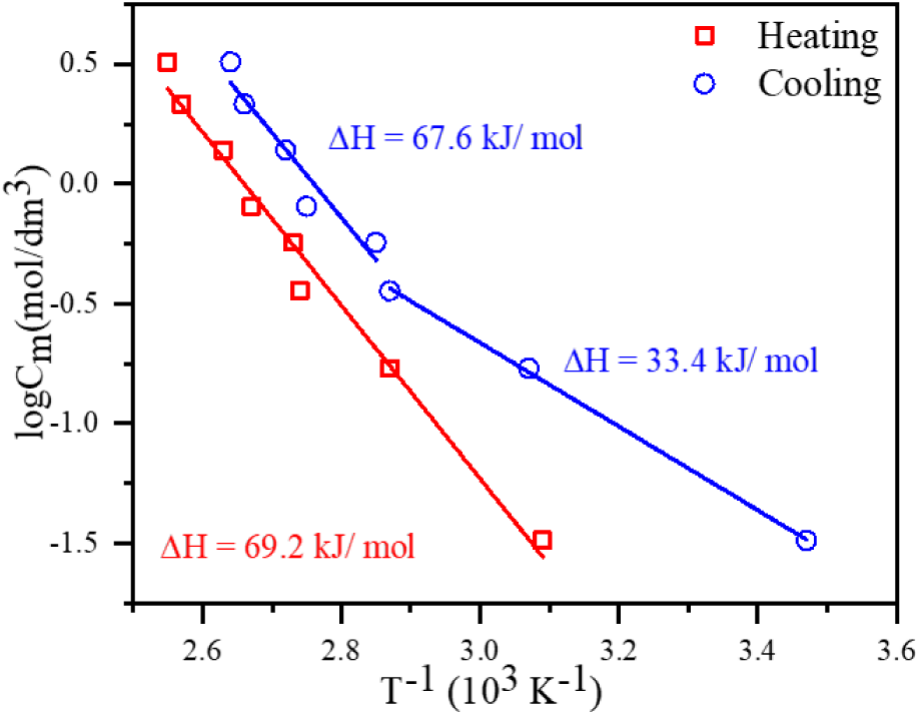
Temperature-dependent *T*_gs_ phase transitions and enthalpy changes associated with the disruption of the gelator network in the studied gel membrane.

To check whether the gel membranes created with gelator concentration above 10 wt % are stable under working conditions at elevated temperatures, we have stabilized the samples at a temperature a few degrees below the maximum of the melting peak for different periods. Afterward, the samples were cooled to room temperature, equilibrated for 30 min, and heated again to 140 °C to observe the melting peak. We have observed a fascinating effect of shifting the maximum temperature of the endothermic peak toward higher temperatures depending on the time and temperature of the preheating stage. In Figure 6, we present the DSC heating curves for 20 wt % gel membrane subjected to and not subjected to thermal treatment. The black curve represents the gel membrane obtained from cooling the hot sol phase to room temperature and not subjected to any other processing procedure. The red curve represents the sample preheated at 100 °C for 1 h before the experiment. Three very well-distinguishable peaks can be observed. The green curve represents the sample subjected to three stages of thermal processing at 100 °C, 110 °C, and 95 °C, stabilized for 1.5 h at each temperature before the experiment. The obtained data show that the internal structure of the gel membrane can be optimized during proper thermal treatment to create a much stronger gelator matrix. The final test of the effect and enhanced gel membrane structure consisted of 10 consecutive cycles of heating the sample to 100 °C and holding it there for 7 h, cooling it to room temperature, and heating it again. The stress test was taken for 75 h; the result is presented in Figure 6. After these extensive working conditions, the membrane retained its gel state and showed the maximum melting peak at 120 °C. We did not observe any influence of the different thermal processing procedures on the exothermic peak related to gelation. Based on this observation, we can conclude that rebuilding the internal gel membrane structure occurs only at elevated temperatures when the weakest interactions are disturbed and reestablished as stronger ones existing in the sample and are not affected by the temperature. The samples were susceptible to thermal processing at all investigated concentrations.

**Figure 6:**
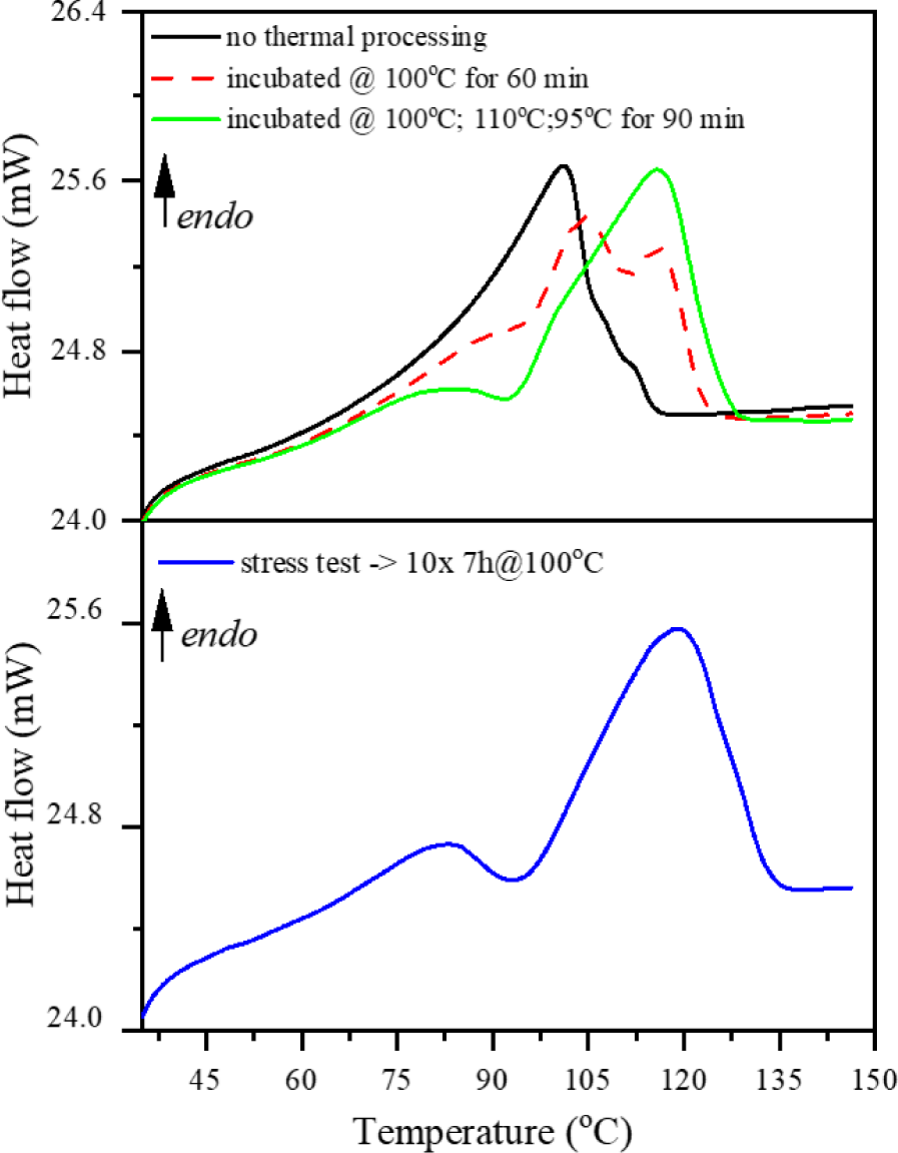
DSC heating curves for G2/PC 20% weight concentration gel membrane, comparing samples before and after the thermal processing stage (upper panel) and the gel membrane after the endurance test (lower panel).

### NMR Investigations

The intermolecular interactions, gelation mechanism, and translational dynamics of the solvent molecules in the liquid and the gel membrane were investigated by using different techniques from the nuclear magnetic resonance method.

Figure 7a presents the ^13^C ssNMR CP-MAS spectrum of gelator G2 and its theoretical spectrum calculated for the optimized molecule conformation. The assignment of the individual lines observed in the experimental spectrum allowed us to identify the crucial regions where changes were observed in the gel membranes. Figure 7b presents the ^13^C NMR spectra for liquid PC acquired under static conditions, solid G2, and the gel membrane acquired under MAS conditions with 10 and 8 kHz of spinning frequency, respectively. The characteristic lines for PC and G2 can be observed in the gel membrane spectrum. The smaller amplitude of G2 concerning PC lines is caused by a low concentration of gelator molecules in the membrane and broader signals from the rigid matrix. The PC lines in the gel membrane spectrum also show a broadening effect that indicates the solvent molecules’ decreased mobility. Recording the carbon spectrum for liquid PC under spinning conditions resulted in no detectable signal due to the high mobility of solvent molecules and ineffective energy transfer in the cross-polarization experiment. This is a consequence of fast dynamics in the liquid state.

**Figure 7:**
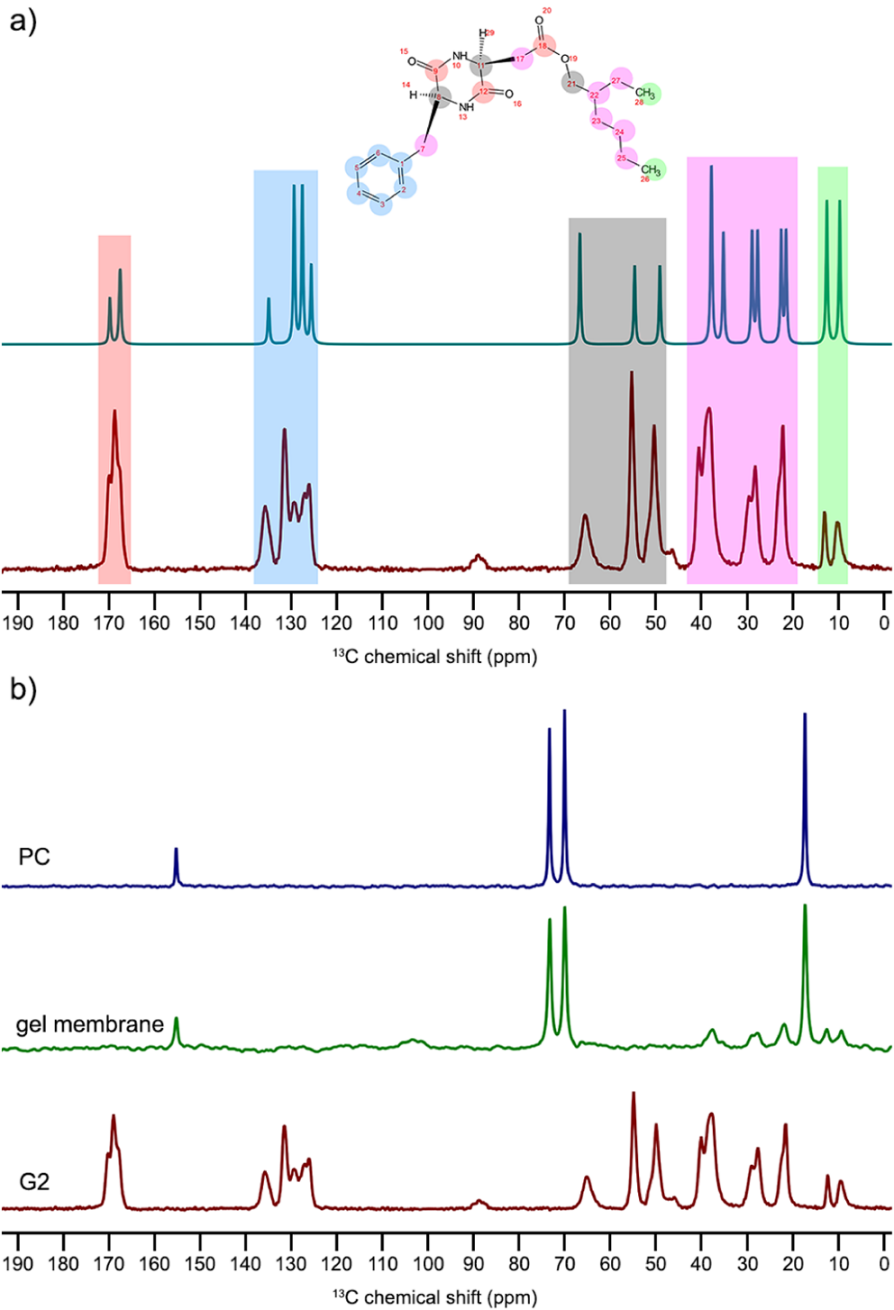
(a) The ^13^C ssNMR CP-MAS spectrum for gelator G2 alongside the theoretical spectrum calculated for the optimized molecular conformation and (b) experimental ^13^C spectra of liquid PC under static conditions, solid-state G2, and 20 wt % G2/PC gel membrane obtained under MAS conditions at 10 and 8 kHz of spinning rate, respectively.

Stronger dipole–dipole interactions in the gel phase allowed the effective use of cross-polarization techniques, and the 2D ^1^H–^13^C HETCOR spectra were recorded for the gel membrane and solid gelator sample. The results are presented in Figure 8. The red spectrum corresponds to G2 in the solid state and green in the gel membrane. Besides additional lines from PC in the gel membrane spectrum, some of the cross-peaks visible in the G2 spectrum in the solid state are missing. The detailed analysis showed that in the case of G2 in solid-state, the energy from hydrogen atoms is transferred over longer distances up to 3 chemical bonds within the molecule compared to the gel membrane under the same measuring conditions. This can be understood if we assume the creation of intermolecular bonds between gelator molecules dispersed in the PC in the gel phase. The lack of energy transferred into the inner part of the G2 molecule and better focusing of cross-peaks in terminal groups suggest that gelator molecules stack in a head-to-tail configuration and create elongated aggregates. Thus, the energy transfer is limited to the terminal groups of the molecules.

**Figure 8:**
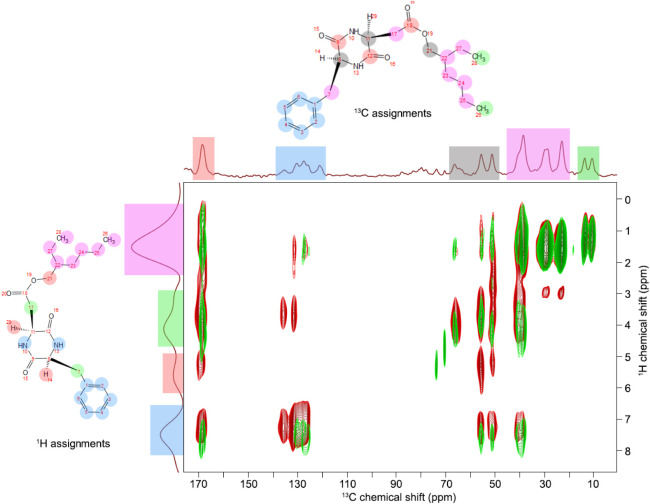
2D ^1^H–^13^C HETCOR spectra for the 20 wt % G2/PC gel membrane (green) and solid-state gelator G2 sample (red).

To investigate how temperature influences the intermolecular interactions between PC molecules, we have recorded the ^1^H NMR spectra as a function of temperature for the liquid state, in the gel membrane state not subjected to thermal processing, and in the gel membrane subjected to a thermal processing procedure. The results are presented in Figure 9 for all three samples. In the case of a thermally processed gel membrane characterized by higher thermal stability, the measurements were continued up to 120 °C to observe the characteristic narrowing of the lines caused by molecules’ increased dynamics upon reaching the liquid state. For the gel membrane sample not subjected to thermal processing and for liquid PC, the highest temperature was 100 °C, which showed characteristic narrowing of the lines in the gel membrane sample. In addition to the change in the line widths, a change in the chemical shifts was observed for the recorded signal. In the case of liquid PC, the chemical shifts of all lines shift toward higher ppm values with increased temperature. However, the shape of the lines is preserved across all temperatures. In the case of the gel membrane not subjected to thermal processing, splitting of the lines can be observed with increased temperature. Consequently, one of the components shifts with temperature analogously to liquid PC, and the second preserves its position up to the gel-to-sol phase transition. Above the *T*_gs_ temperature, the static component vanishes. In the case of thermally processed gel membranes, the situation is different. Starting from room temperature, the signals show at least two components that merge into one broad signal with increased temperature. Further increases in temperature, however, did not cause the shift of the lines displaying the same behavior as one of the two components recorded for the thermally unprocessed gel membrane. Based on the observation for both gel samples, we can conclude that the components of the lines which do not change the chemical shift are related to the PC molecules interacting with the gel matrix, whereas the components that shift their position to the PC molecules within the vacancy of the pores, not interacting with the matrix surface. The molecule that does not interact with the matrix behaves as in the bulk liquid state. In the thermally processed gel membrane, the only evidence of the gel-to-sol phase transition is the narrowing of the line widths above the *T*_gs_ temperature.

**Figure 9:**
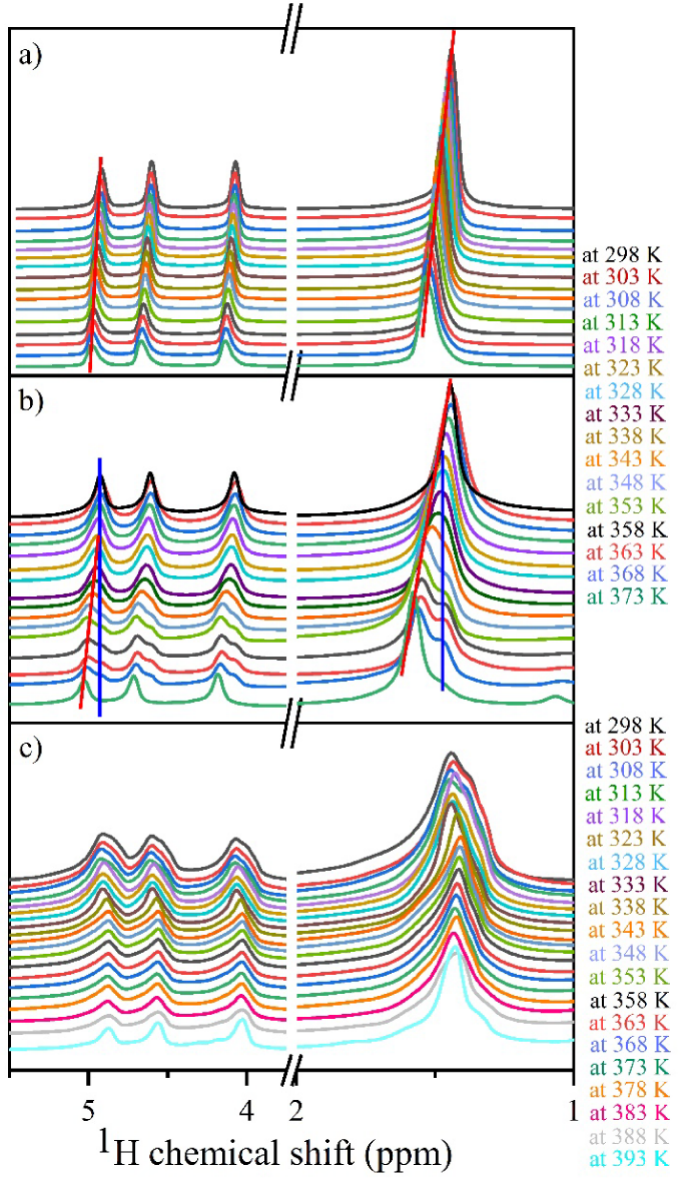
^1^H NMR spectra of propylene carbonate (PC) as a function of temperature for a pure liquid-state sample (a), in the G2/PC 20 wt % gel membrane state before thermal processing (b), and after the thermal processing (c) stage. The blue and red lines are eye-guidelines for visualizing chemical shifts for noninteracting (red) and interacting (blue) PC molecules with the gel matrix.

To study the translational dynamics of the immobilized solvent molecules, we performed the ^1^H pulse field gradient (PFG) experiments as a function of temperature for PC in the liquid state and within the gel membrane. As a result, the self-diffusion coefficients were determined for both systems. The temperature dependences of the self-diffusion coefficients for PC molecules are presented in Figure 10.

**Figure 10:**
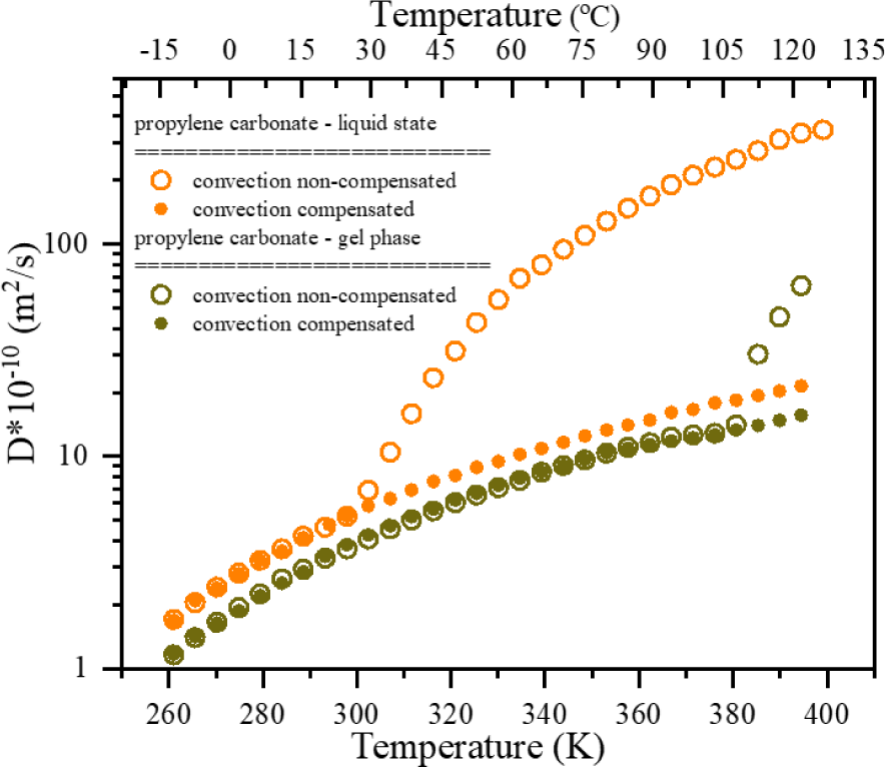
Temperature dependence of self-diffusion coefficients for PC molecules in the pure liquid state (orange) and gel membrane state (green).

As a consequence of elevated temperatures and a temperature gradient within the samples, the effect of thermal convection on liquid PC was observed. The thermal convection causes additional bulk flow, which attenuates the signal, leading to higher values of the self-diffusion coefficients. We have applied a double-stimulated-echo pulse sequence (DSTE) to compensate for this unwanted effect. The results of DSTE measurements are presented as solid symbols in Figure 10. As can be seen, this approach completely removed the convection effect up to the highest temperatures. The measurements for the gel membrane showed that the convection in the membrane is naturally eliminated by immobilizing the solvent molecules within the gel matrix up to the gel-to-sol phase transition temperature (*T*_gs_). Above the *T*_gs_, the internal gelator matrix is disrupted, and the bulk flow caused by thermal convection starts to attenuate the signals, resulting in a substantial increase in the observed self-diffusion coefficient. Again, using the DSTE pulse sequence allowed us to eliminate the convection effect.

Another important fact is that the absolute diffusion coefficient for the translational motion of PC molecules in the liquid and gel phases is of the same magnitude. This indicates that the gel matrix does not significantly reduce the microscopic mobility of the solvent molecules yet prevents macroscopic bulk flow. To investigate if the gel matrix in the membrane causes geometrical restrictions to PC’s translational motion, the measurements were performed as a function of so-called diffusion time (Δ). Figure 11 shows the self-diffusion coefficients for solvent molecules in liquid form, the gel membrane not subjected to thermal processing, and the gel membrane subjected to thermal processing (displaying enhanced thermal stability). When diffusing molecules do not experience geometrical obstacles in their way, the self-diffusion coefficient is not dependent on the diffusion time; the molecules are in a so-called free diffusion regime. When the geometrical restrictions start to play a role, the self-diffusion coefficient decreases with the increase in Δ, and we observe so-called restricted diffusion. The measurements performed at room temperature indicate that the gel membranes’ internal matrix does not cause geometrical restrictions within the observed diffusion time range.

**Figure 11:**
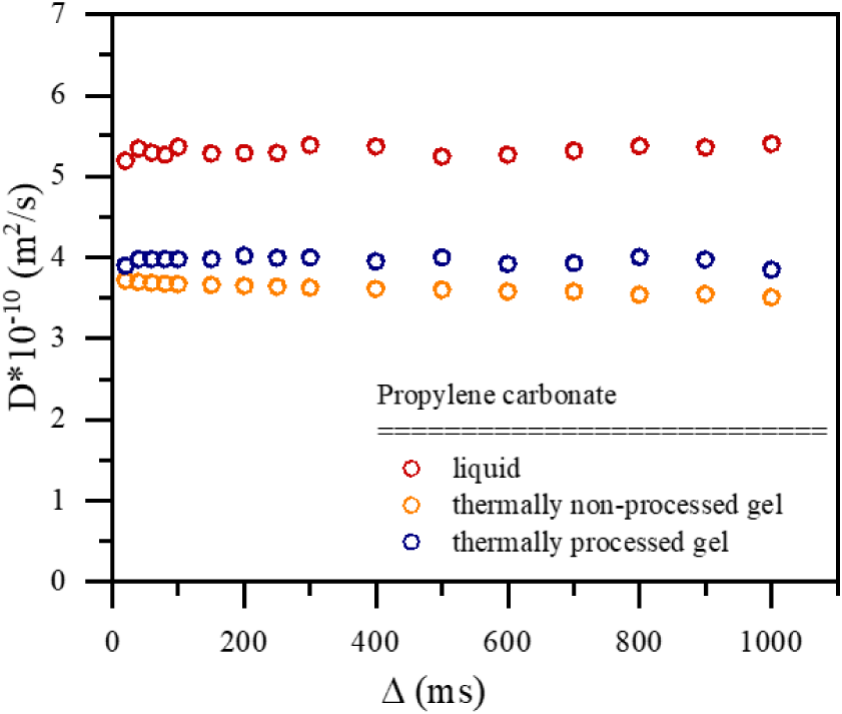
Self-diffusion coefficients *D* of propylene carbonate in liquid form (red) and 20 wt % G2/PC gel membranes before thermal processing (orange) and after thermal processing (blue) state studied as a function of the diffusion time Δ.

To check the character of the translational motion of the PC molecules, we have analyzed the activation energies for this motion in the liquid and gel membrane states. The results are presented in Figure 12. For the calculation of the activation energies, we used the Arrhenius equation for thermally induced processes:

2 $$k=Ae\frac{-E_{a}}{RT}$$

**Figure 12:**
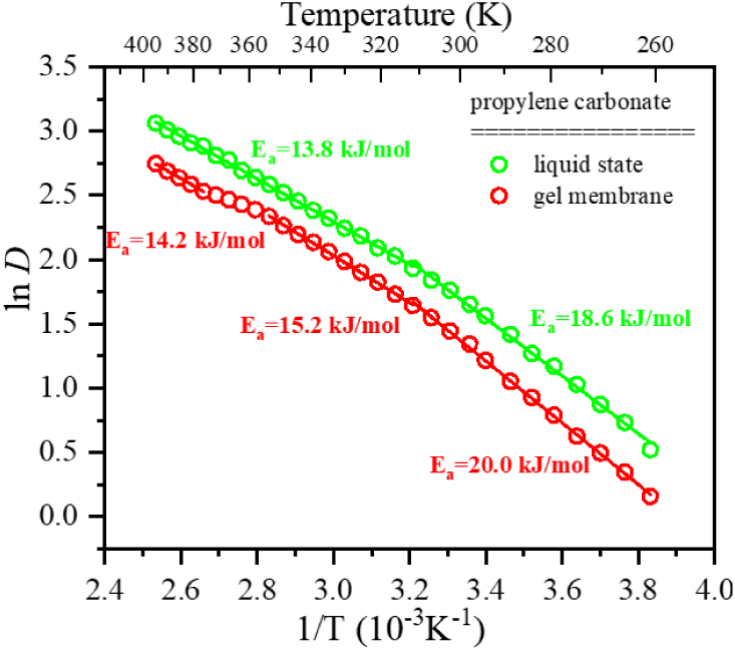
Activation energies for the translational motion of PC molecules in the pure liquid state (green) and gel membrane before and after the gel-to-sol phase transition (red).

The straight lines in Figure 12 show the best fits of eq 2 to the experimentally obtained self-diffusion coefficients.

The analysis has shown that in liquid PC, two *E*_a_ have been determined in the temperature range from −25 to 40 °C and second from 40 to 130 °C, equal to 18.6 and 13.8 kJ mol^–1^, respectively. The propylene carbonate molecules are known to create head-to-tail aggregates through hydrogen bond interactions. The strength of these bonds is generally inversely proportional to the temperature. Therefore, at low temperatures, the PC molecule aggregates are bigger and more strongly connected. At higher temperatures, the intermolecular H-bonds weaken, and the aggregates become smaller, which implies lowering the activation energy for translation motion. In the case of the gel membrane sample, the situation is similar only in that *E*_a_ is higher concerning liquid PC, and we observe one more activation energy for the temperature range above the gel-to-sol phase transition. Within the whole temperature range of the gel phase, the *E*_a_ is 1.4 kJ mol^–1^ higher than that for liquid PC and becomes only 0.4 kJ mol^–1^ higher above the gel-to-sol phase transition. This behavior proves that the gelator matrix and interactions on its surface are responsible for increasing the activation energy for translational motion. After melting the matrix and transforming the gel membrane into a liquid phase, the activation energy of the translational motions becomes very similar yet higher to that of the pure liquid PC solvent. The small extent of *E*_a_ in the hot sol phase can be related to the increased viscosity of the G2/PC mixture concerning pure PC liquid.

### Scanning Fluorescence Confocal Microscopy Analysis (SFCM)

The internal structure of the gel matrix plays a crucial role in the gel membrane’s thermal stability, translational mobility, and elimination of thermal convection.

To get insight into the microstructure of the studied systems, we used SFCM. This method allows us to investigate the supramolecular gels containing the solvent phase without the danger of locally overheating the system and evaporating the solvent, which can happen using other techniques of higher resolution, such as TEM or SEM. Using the Gwyddion software package and statistical analysis, we determined the distribution of the length and width of the LMWG aggregates and pores. The results are presented as distribution charts in Figures 13 and 14 for 10% and 50% weight concentration gel membranes, along with corresponding SFCM images.

**Figure 13:**
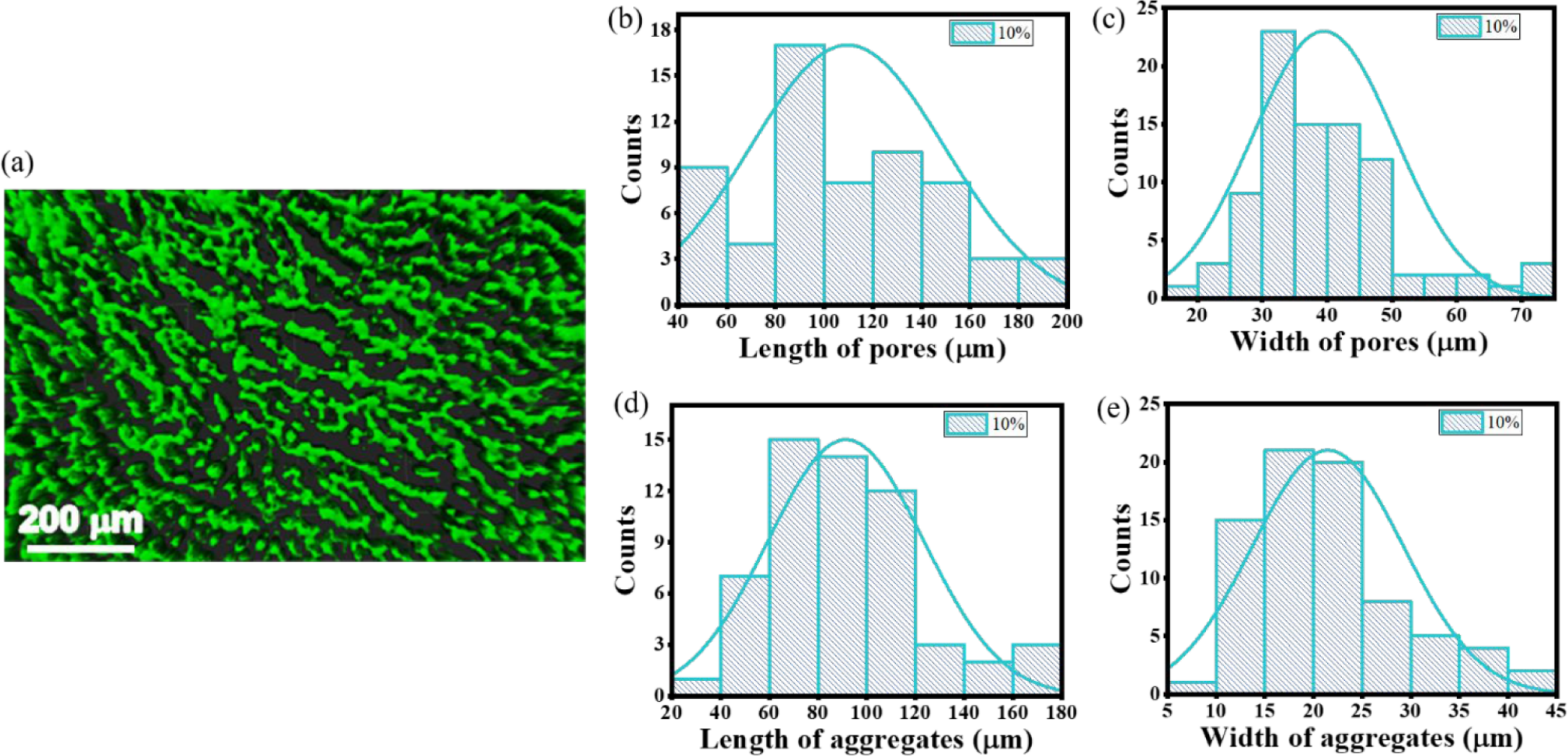
SFCM image (a) of the microstructure of the 10% gel membrane and statistical analysis (b–e) of LMWG aggregate widths and pore sizes along the long and short axes.

**Figure 14:**
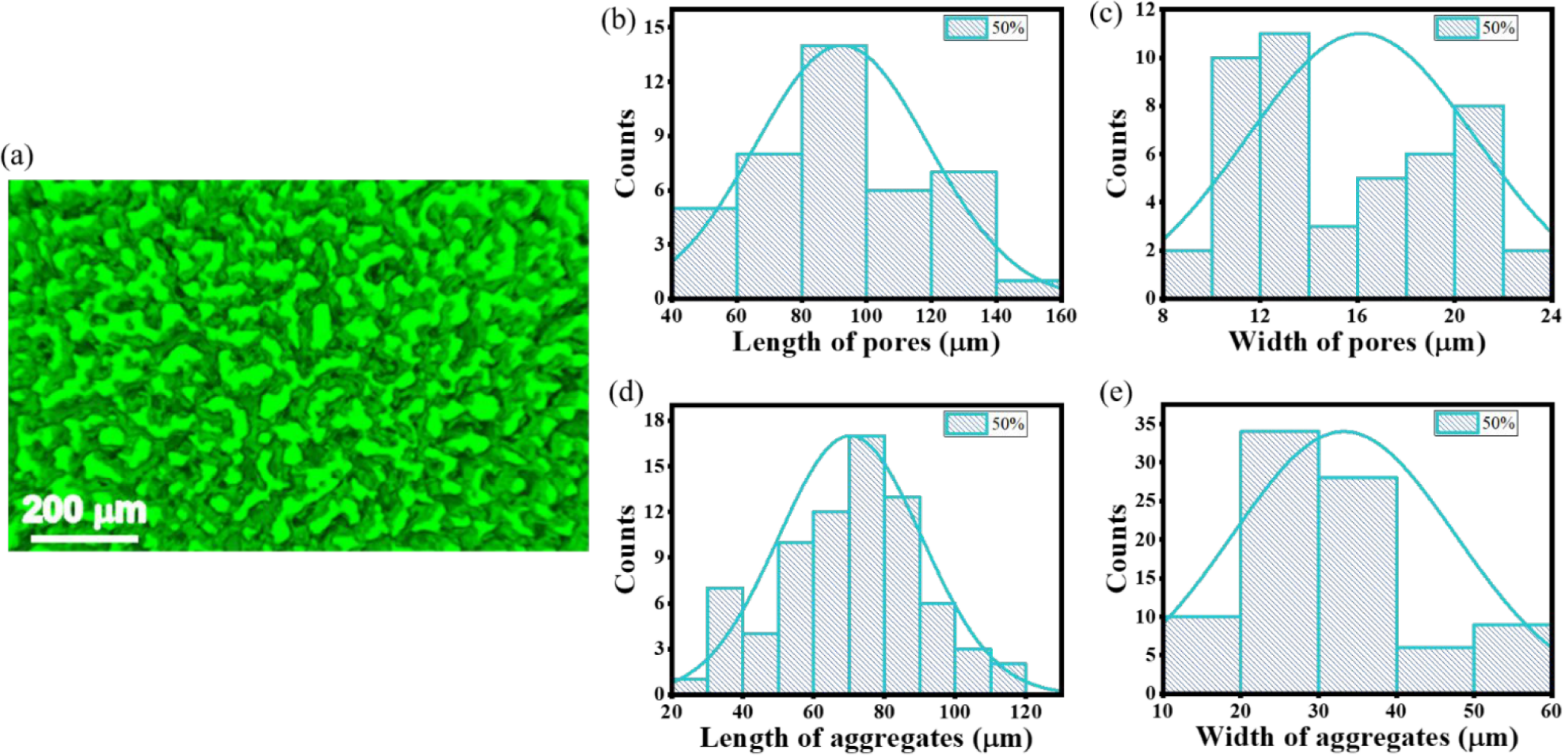
SFCM image (a) of the microstructure of the 50% gel membrane and statistical analysis (b–e) of LMWG aggregate widths and pore sizes along the long and short axes.

As can be seen, the increase in gelator concentration has affected the self-aggregation process and led to an increase in the aggregates’ width, leaving the length of aggregates at a similar size. Accordingly, pore size changes were observed when the gelator content was increased. The width of the pores decreased, but the length stayed roughly the same. However, the observed changes are minor concerning the significant increase in the LMWG content.

With the increase of the gelator concentration, the thickness of created aggregates forming a branching fiber structure at low concentrations evolves into a much thicker mosaic-like structure at high concentrations. The free spaces between G2 aggregates, forming pores and channels within which the solvent molecules move, remain the same size but become more tortuous. As a consequence, the thermal stability of the matrix increases, and the size of the interconnected porous structure does not restrict the translational motion of the liquid phase. The average size of the pores in the shorter dimension stays at a level from a dozen to several dozen micrometers.

### Rheology Studies

To investigate the viscoelastic properties and confirm the gel behavior of G2 systems, oscillatory rheological tests were performed on samples at concentrations of 2%, 10%, and 20% w/w. Amplitude sweep measurements (0.01–100% strain at 1 Hz) and frequency sweep tests (0.1–100 Hz at 0.1% strain) were conducted at 25 °C (G2 2%), 80 °C (G2 10%), and 120 °C (G2 20%).

As shown in Figure 15, the amplitude sweep results reveal a well-defined linear viscoelastic region (LVE) for all samples, with *G*′ remaining constant up to ∼1% strain (Figure 15a). Within this region, the storage modulus (*G*′) consistently exceeds the loss modulus (*G*″), indicating a predominant elastic response and the presence of a gel-like network. Beyond the LVE, a crossover between *G*′ and *G″* signifies the onset of yielding and structural breakdown of the gel (Figure 15b). Noteworthy, despite being measured at elevated temperatures (80 °C for 10% and 120 °C for 20%), the *G*′ values were still significantly higher than those of the 2% sample at room temperature (25 °C). This observation suggests that concentration exerts a stronger influence than temperature on the mechanical strength of the gel matrix.

**Figure 15:**
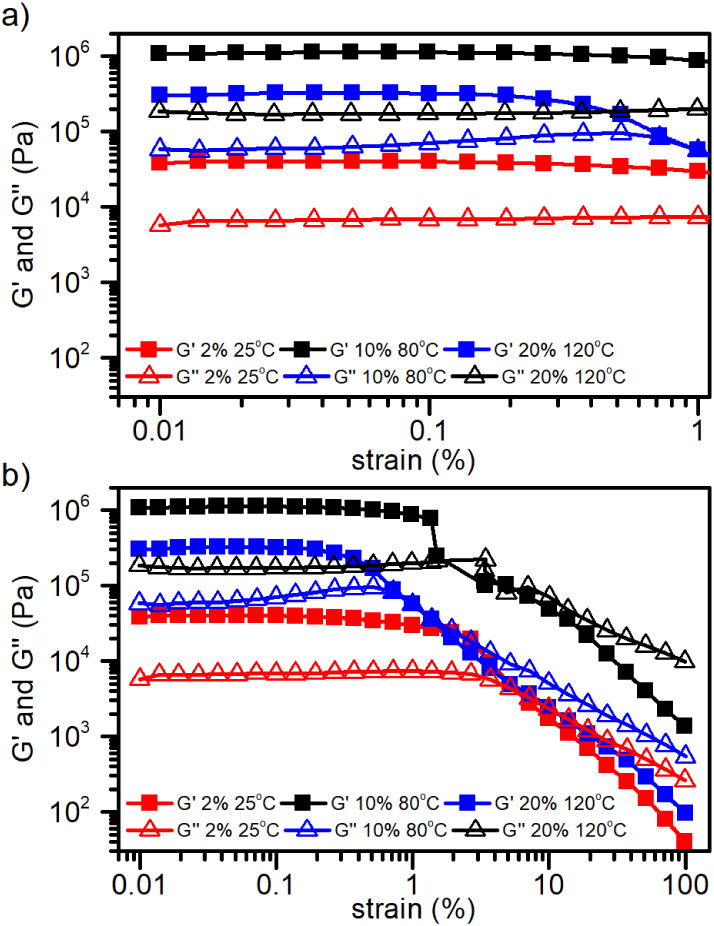
Rheological measurements on G2 gels (2%, 10%, and 20%)—amplitude-sweep measurement at a constant frequency of 1 Hz. (a) Strain-sweep from 0.01 to 1% strain. (b) Strain-sweep from 0.01 to 100% strain.

Figure 16 presents the frequency sweep results, which further corroborate the gel-like nature of all G2 samples. Throughout the entire frequency range, *G*′ remains greater than *G*″, and both moduli show weak frequency dependence, consistent with solid-like gel behavior. Additionally, a clear temperature-dependent trend also emerges: while higher concentration gels exhibited increased stiffness, the elevated measurement temperatures reduced brittleness and enhanced compliance, particularly in the 20% sample. This sample could not be reliably measured at room temperature due to excessive brittleness and fracture under minimal deformation (exceeding 50 N of normal force). However, at 120 °C, the material exhibited measurable viscoelastic properties, indicating a plasticizing effect of the temperature on the gel network.

**Figure 16:**
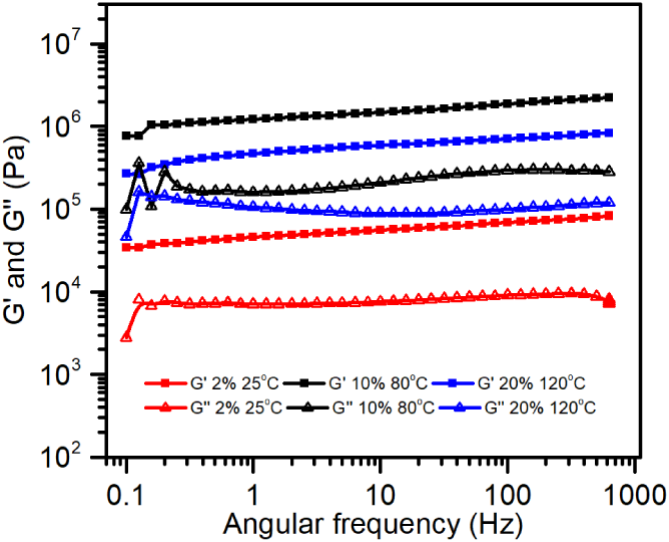
Evolution of *G*′ and *G*″ as a function of the angular frequency (ω).

## Experimental Section

### Preparation of the Gel

The low molecular weight gelator l-β-2-ethylhexylasparaginyl-l-phenylalanine (G2) was synthesized by Synthex Technologies Sp. z o.o., Toruń, Poland on special order (the spectroscopic and purity characterization of synthesized G2 molecules was confirmed by spectroscopic methods. The results are presented in Figures S1–S4). The molecular structure of this gelator has been proposed and synthesized for the first time by Hanabusa et al. as a specialized gelator for ionic liquids.^26^ Propylene carbonate (PC) was purchased from Merc Co. and used without further processing. The investigated supramolecular gel samples were prepared according to the following procedure. The gelator (G2) was dissolved in liquid PC upon heating and stirring the mixture in closed-cap glass vials at 120 °C for 15 min. After the complete dissolving of solid G2, the mixture was heated and stirred further for 5 min at 120 °C to obtain a homogeneous sol phase. Afterward, the sample was left to cool at room temperature without stirring; during this stage, the sol-to-gel phase transition occurred. To investigate the efficiency of the gelation process, the CMC was determined by preparing low concentrations of G2 in PC and checking the time required by the system to immobilize the solvent and its ability to prevent bulk flow in the reverse tube test. The following series of gel samples at 1%, 5%, 10%, 15%, 20%, 30%, 40%, and 50% weight concentrations of G2 in 2 mL of PC was prepared. The pure liquid PC and solid-state G2 were used as reference systems. The sample passed the reverse tube test if, after 10 min of cooling at room temperature, it prevented bulk flow for at least 30 min. The sample failed the test if it could not stop the bulk flow regardless of the gelation time. Based on these assumptions, the CMC was determined to be 2 wt % of G2 in PC (Figure S5). The gel exhibited a visually opaque appearance and demonstrated exceptional stability over an extended duration, exhibiting no observable alterations or phase separation for months. The preparation process for the LMWG-based gel is illustrated in Figure 17.

**Figure 17:**
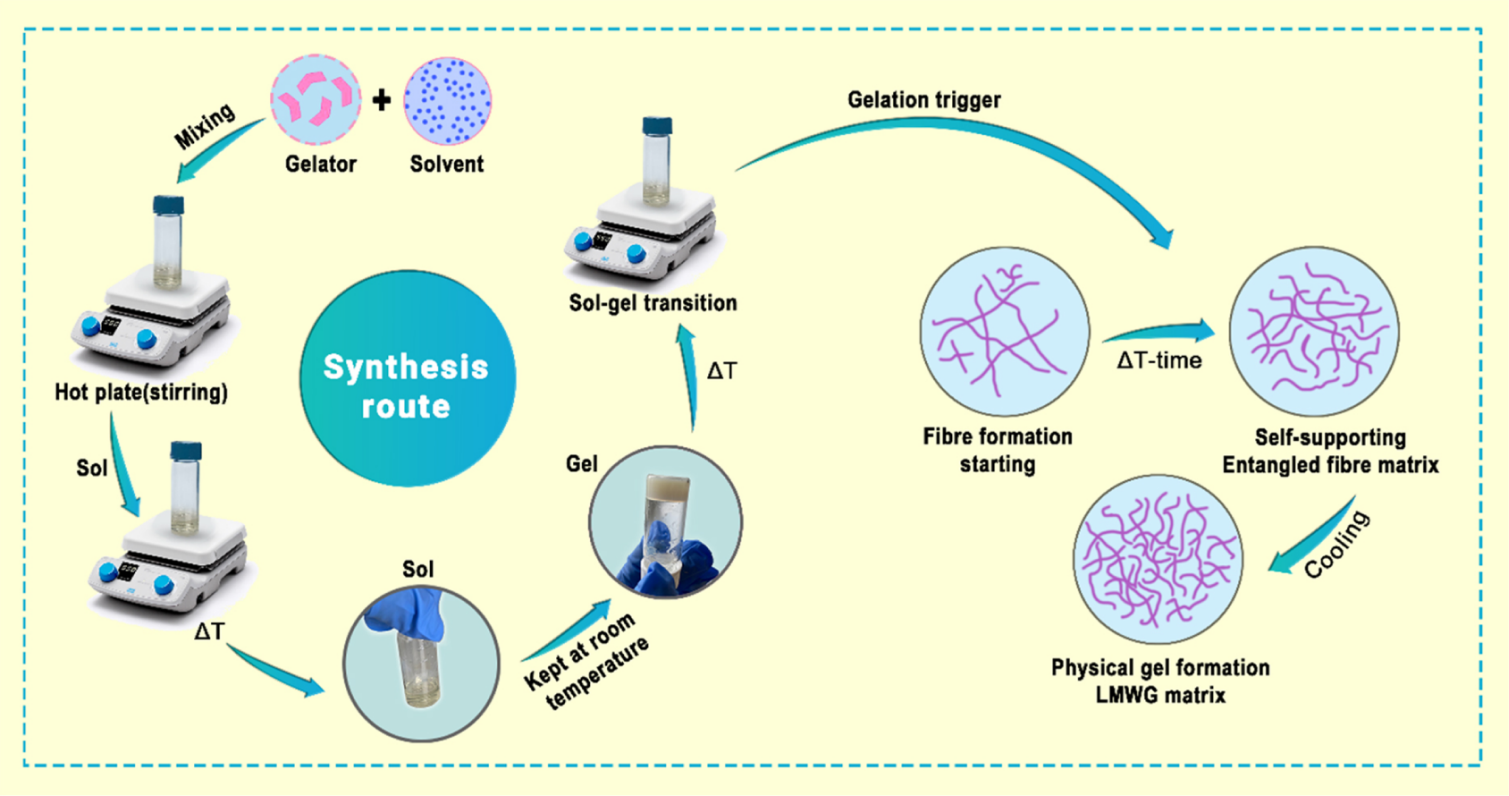
Preparation route of G2/PC gel membrane systems with a postpreparation thermal processing stage for structural enhancement.

### Thermal Analysis Methods (TGA/DSC)

The thermogravimetry method (TGA) was used to analyze the sample’s mass loss upon thermal degradation during the heating stage. The measurements were performed on a PerkinElmer TGA8000 analyzer under a nitrogen atmosphere. Samples were placed in a platinum heating pan. The heating rate was equal to 10 °C/min, and all samples were investigated in the 20–600 °C temperature range, which allowed us to observe the decomposition of all components. The results were analyzed according to the derivative method (DTG).

The phase behavior of the investigated samples was analyzed with the differential scanning calorimetry (DSC) method on a PerkinElmer DSC4000 analyzer. The measurements were performed in subsequent heating–cooling cycles in a nitrogen atmosphere at a temperature change rate of 10 °C/min in the temperature range from 30 to 140 °C except for the lowest concentration, which required the temperature range of 0 to 140 °C. The samples were carefully closed and sealed in aluminum crucibles able to hold 3 bar of internal pressure. Three consecutive heating and cooling cycles were recorded for each sample to confirm the reversibility of gel-to-sol and sol-to-gel phase transitions detected as endothermic and exothermic events in thermograms.

### Nuclear Magnetic Resonance Methods (NMR)

The various NMR methods were used to investigate the intermolecular interactions between the components of studied supramolecular systems and examine the solvent molecules’ translational dynamics at the gel state. The 1D and 2D high-resolution ssNMR techniques ^1^H–^13^C CP-MAS, ^13^C DP-MAS, and ^1^H–^13^C HETCOR, together with spectral editing sequences, were used to investigate the composition of obtained systems and intermolecular interactions between the components in different states. The NMR diffusometry method was used to investigate the ^1^H NMR spectra in the gel, sol, and liquid phases to determine the influence of the created 3D gel matrix on solvent molecules’ translational dynamics in the confinement state. The experiments were performed on a Bruker Avance III HD NMR spectrometer coupled to an 11.4 T superconducting magnet operating at 125.76 MHz for carbon Larmour frequency. The temperature of samples was controlled and stabilized using a Bruker VTC at 0.2 °C accuracy. For high-resolution ssNMR measurements, a spinning frequency of 10 kHz was used for the solid-state gelator sample and 8 kHz for the gel sample. The gel samples were placed in unique 25 μL Kel-F inserts before measurements. The lower spinning rate and special inserts were used to avoid the danger of mechanically destroying the gel state due to excessive centrifugal force and leakage of the solvent.

Cross-polarization (CP) techniques were used to improve the S/N ratio of the recorded signals and reduce the total time of a single experiment. The measurements were performed with contact time set to 1500 μs, the recycle delay was set to 6s, and 2048 scans were accumulated, resulting in over 3 h of acquisition time per spectrum. For direct-polarization (DP) experiments, the recycle delay was set to 10 s, and 4096 scans were accumulated, resulting in over 11 h of acquisition time per spectrum.

The diffusion NMR experiments were performed using a Diff50 probe from Bruker, allowing to apply magnetic field gradients of maximum strength 3000 Gs/cm. The samples were placed in closed-cap 5 mm NMR tubes. The stimulated echo (STE) and double stimulated echo (DSTE) sequences in the pulse field gradient (PFG) experiment were used to determine the self-diffusion coefficient of solvent molecules in the gel, sol, and liquid states. The recycling delay was set to 10 s, and 8 scans were accumulated, resulting in 45 min of acquisition time per single experiment. The measurements were conducted as a function of temperature from 263 to 398 K and as a function of so-called diffusion time Δ ranging from 20 to 1000 ms.

### Scanning Fluorescence Confocal Microscopy Analysis (SFCM)

The fluorescence confocal scanning microscope (Olympus Fluoview 1000 IX81) was used to investigate the microstructure of gel membranes. We have used the diode lasers with wavelengths of λ = 405 and λ = 473 nm as a source of the excitation beam. 46% and 34% of the maximum laser power were applied to the tested materials, respectively. The fluorescence signal was collected in a wide spectral range, from 490 to 635 nm. To obtain a three-dimensional image, the sample was scanned in the *XY* plane with a focused beam, which changed the confocal plane in the *Z* direction. Each scan in the horizontal plane was repeated five times, and the obtained image was the average of these measurements. The thickness of the sample from which the signal was collected in the XY plane was 2.66 μm.

### Rheology Study

The oscillatory shear rheological analysis was conducted at temperatures of 25, 80, and 120 °C for 2, 10, and 20%, respectively, using an Anton Paar rotational rheometer (Graz, Austria) equipped with parallel plate geometry, featuring a 25 mm diameter and a 0.5 mm gap. Around 1.0 ± 0.2 g of a freshly prepared gel sample was placed evenly on the bottom plate. To identify the linear viscoelastic region (LVE) of all samples, initial dynamic strain amplitude sweep tests were performed in the 0.01–100% range at a constant frequency of 1 Hz, monitoring the storage modulus *G*’. Following this, dynamic frequency sweep measurements (0.1–100 Hz) were carried out for all samples at a strain value of 0.1%, as determined in the strain sweep test. In order to study the flow and deformation properties of gels, oscillatory rheological measurements (amplitude-sweep tests) were initially conducted on gels (2, 10, and 20 wt %). It is evident that, for all of the aforementioned gels, the elastic component (*G*′) exerts a dominant influence over the viscous component (*G*″) at low levels of applied shear. Furthermore, it is observed that *G*′ reaches a plateau within the linear response viscoelastic region (LVR).

## Conclusions

In summary, we have demonstrated that the l-β-2-ethylhexylasparaginyl-l-phenylalanine LMWG can self-assemble in propylene carbonate at various weight concentrations of the gelator molecules ranging from 1 to 50 wt %, creating rigid matrices able to immobilize large quantities of liquid molecules. Obtained DSC results have shown that the solidification process is thermally reversible and that supramolecular structures are susceptible to thermal processing. This leads to enhanced thermal stability and durability of the created gel-like membranes. The structural enhancement is caused by the reorganization and reconstruction of G2 aggregates, created initially during the sol–gel phase transition upon cooling the hot sol sample, which was exposed to elevated temperature for 90 min just below the initial gel-to-sol phase transition temperature. The investigations of intermolecular interactions and translational dynamics have shown that the motion of solvent molecules on a microscopic scale is not significantly affected by the presence of the solid matrix, leading to self-diffusion coefficients in the membranes of the same order of magnitude as in liquid PC, yet effectively preventing bulk flow on a macroscopic scale. The intermolecular interactions at the surface of the matrix, evidenced by the splitting of the lines in the ^1^H NMR spectrum of PC and broadening of the lines, influence the nearest solvent molecules, and the range of the influence depends on the supramolecular microstructure. This interaction has a more extended range of influence in thermally processed membranes. The increase of the gelator concentration in membranes leads to a rise in the size of the G2 aggregates forming the matrix and a change of its shape, creating a tortuous porous system in which the minimal dimension of free spaces is in the range of a dozen micrometers. Based on the rheology studies, we can say that the membrane phase is strongly dependent on the configuration of the gelator and transforms from gel-like behavior to solid-like behavior preserving high mobility of the embedded liquid phase. The determined enthalpies of endothermic and exothermic phase transitions revealed by DSC showed two regimes, indicating that systems with 10 wt % and above of the gelator content display efficient self-healing properties, increasing the membranes’ durability. The determined activation energies for the translational diffusion showed that the presence of the matrix uniformly influenced this energy by increasing it by 1.5 kJ mol^–1^ with respect to liquid PC. Additionally, we have shown that the supramolecular membranes eliminate the unwanted thermal convection effect that strongly influences transport properties. Therefore, the created membrane systems have a high potential to be used as alternatives for solidification processes, allowing for the elimination of bulk flow, retention of the shape and size, easy recovery process, and thermal stability for materials transformed into solid form from a liquid state.

## Supplementary Material



## Notes

^1^H NMR spectra of cyclo­(l-β-2-ethylhexylasparaginyl-l-phenylalanyl) in DMSO; chromatogram of gelator; MS chromatogram and MS spectrum of gelator; and gel images of G2 in propylene carbonate at varying concentrations (1% below MGC, 2% at MGC, 50% above MGC) (PDF)

## Abbreviations

LMWG: low molecular weight gelator
PC: propylene carbonate
*T*_gs_: gel-to-sol phase transition temperature
SFCM: scanning fluorescence confocal microscopy
DSTE: double-stimulated-echo pulse sequence
PFG: pulse field gradient
