# Supplementary material for: Translational Dynamics and Structural Enhancement Effect in High-Temperature Supramolecular Systems of Asparaginyl Low-Molecular-Weight Gelators and Propylene Carbonate
Source: Macromolecules. 2025 Sep 5;58(18):9786–98. doi: 10.1021/acs.macromol.4c03225 (PMC12462232; doi:10.1021/acs.macromol.4c03225)
Supplement: Supplementary file 1 [file ma4c03225_si_001.pdf]

Supporting Information

Translational dynamics and structural enhancement effect in high-temperature supramolecular systems of asparaginyl low molecular weight gelator and propylene carbonate

Farooq Ahmad[a], Natalia Bielejewska[a], Dawid Pakulski,[b] and Michał Bielejewski[a]*

[a]Institute of Molecular Physics, Polish Academy of Sciences,
M. Smoluchowskiego 17, Poznań 60-179, Poland.
[b]Centre for Advanced Technologies, Adam Mickiewicz University,
Uniwersytetu Poznańskiego 10, Poznań 61-614, Poland

KEYWORDS: Supramolecular Gel; Translational dynamics; Thermal analysis; NMR spectroscopy; Phase transition temperature

AUTHOR INFORMATION

Corresponding Author

***Michał Bielejewski** - *Institute of Molecular Physics, Polish Academy of Sciences, M. Smoluchowskiego 17, Poznań 60-179, Poland.* Email: bielejewski@ifmpan.poznan.pl

Authors

Farooq Ahmad - *Institute of Molecular Physics, Polish Academy of Sciences, M. Smoluchowskiego 17, Poznań 60-179, Poland.* Email: farooq.ahmad@ifmpan.poznan.pl

Natalia Bielejewska - *Institute of Molecular Physics, Polish Academy of Sciences, M. Smoluchowskiego 17, Poznań 60-179, Poland.* Email: natalia.bielejewska@ifmpan.poznan.pl

Dawid Pakulski - *Centre for Advanced Technologies, Adam Mickiewicz University, Uniwersytetu Poznańskiego 10, Poznań 61-614, Poland*. Email: dawid.pakulski@amu.edu.pl

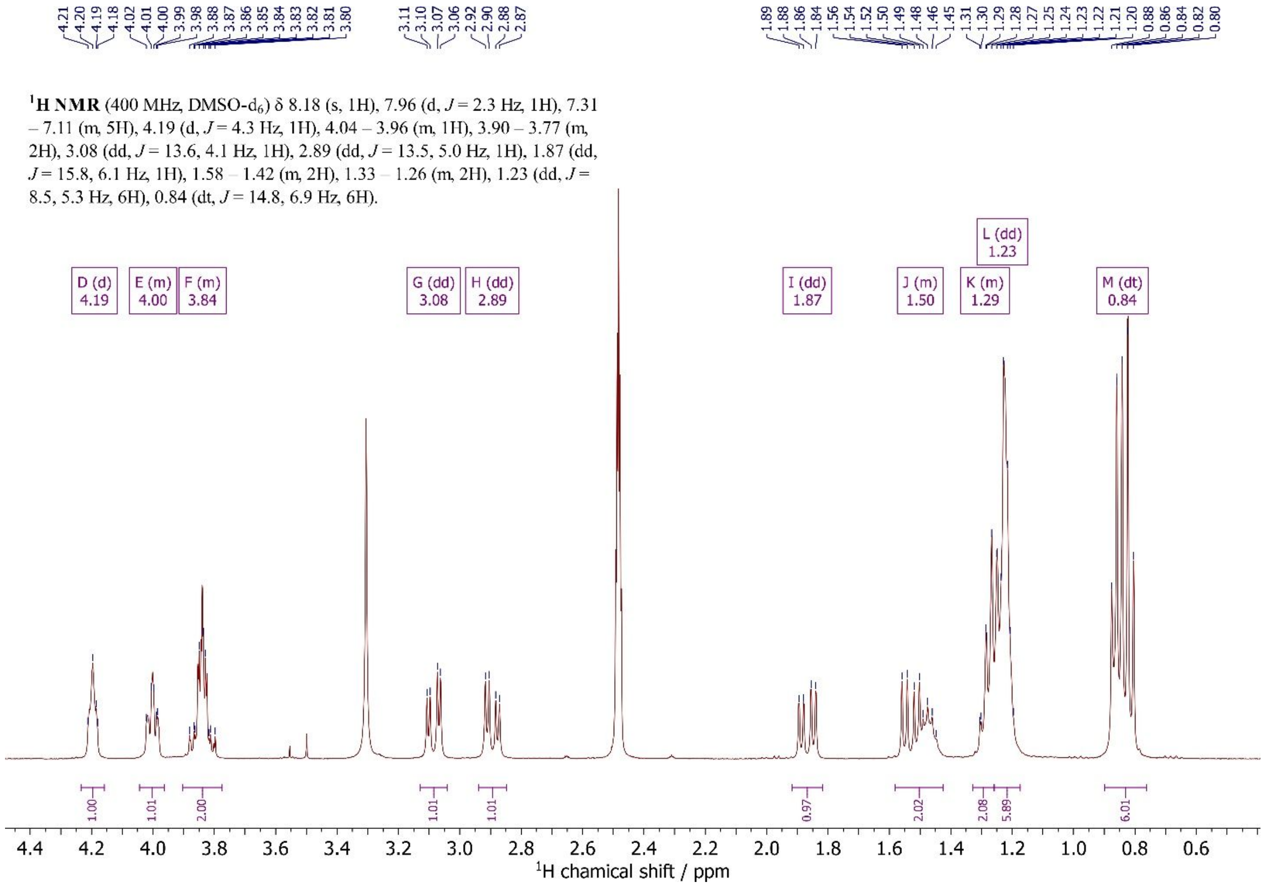

Figure S1. ^{1}H NMR spectra of the gelator cyclo(L-beta-2-ethylhexylasparaginyl-L-phenylalanyl) in DMSO.

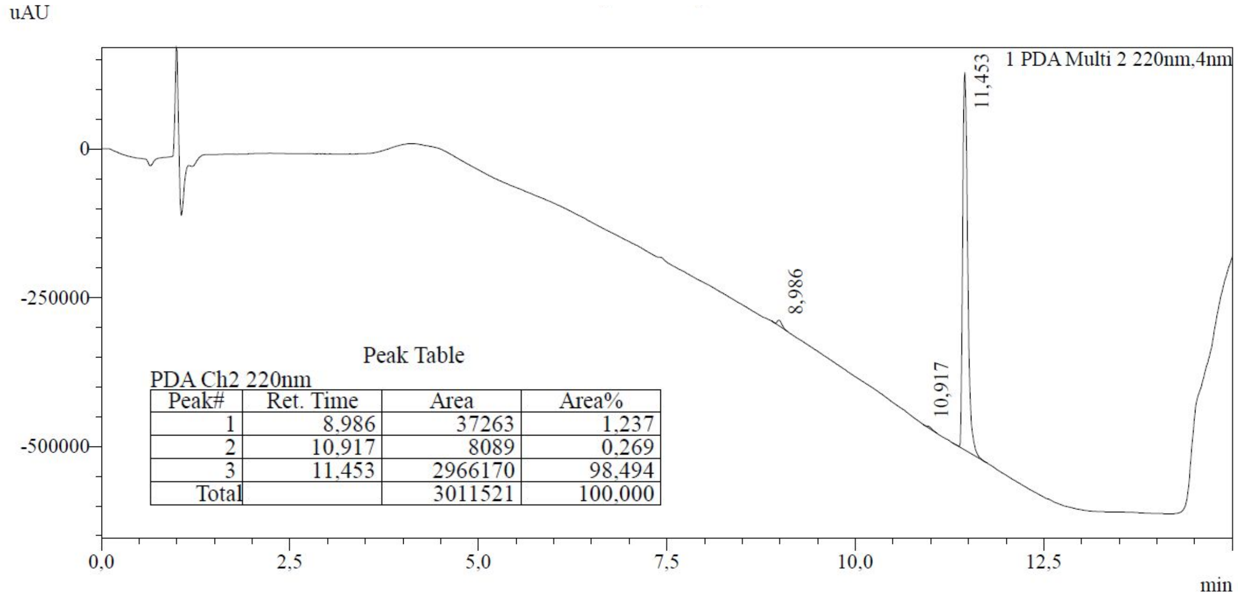

Peak Table

PDA Ch2 220nm

Peak#	Ret. Time	Area	Area%
1	8,986	37263	1,237
2	10,917	8089	0,269
3	11,453	2966170	98,494
Total		3011521	100,000

Figure S2. Chromatogram of the gelator cyclo(L-beta-2-ethylhexylasparaginyl-L-phenylalanyl).

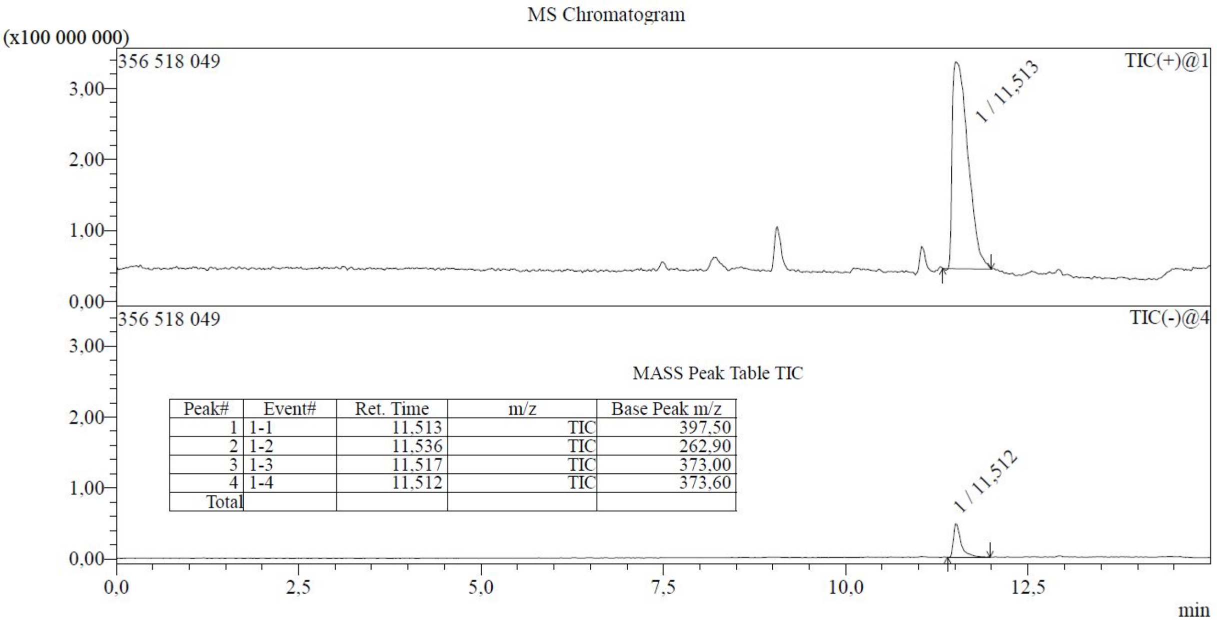

Peak#	Event#	Ret. Time	m/z	Base Peak m/z
1	1-1	11,513	TIC	397,50
2	1-2	11,536	TIC	262,90
3	1-3	11,517	TIC	373,00
4	1-4	11,512	TIC	373,60
Total				

Figure S3. MS chromatogram of the gelator cyclo(L-beta-2-ethylhexylasparaginyl-L-phenylalanyl).

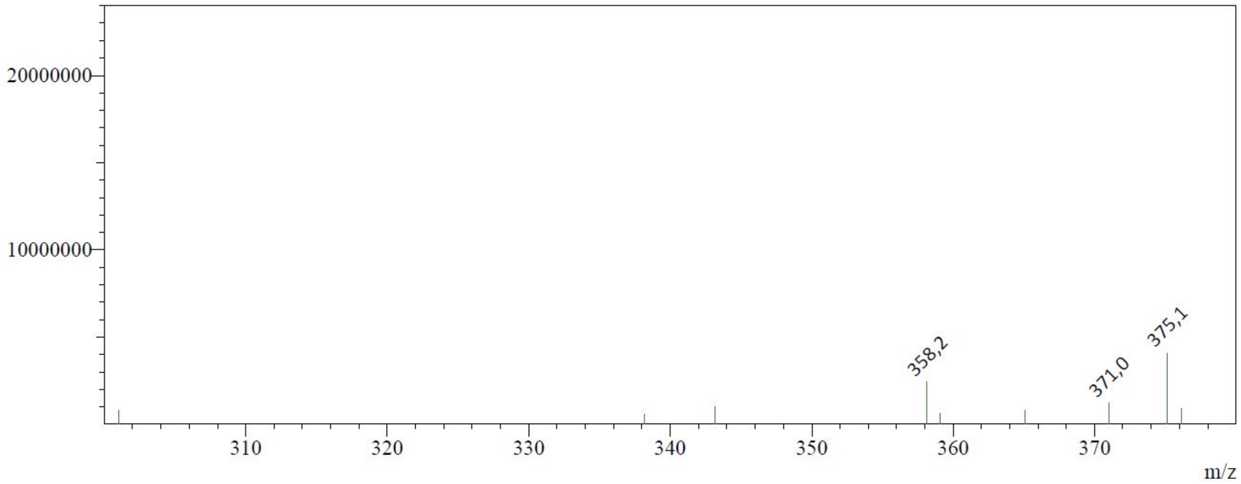

Figure S4. MS spectrum of the gelator cyclo(L-beta-2-ethylhexylasparaginyl-L-phenylalanyl).

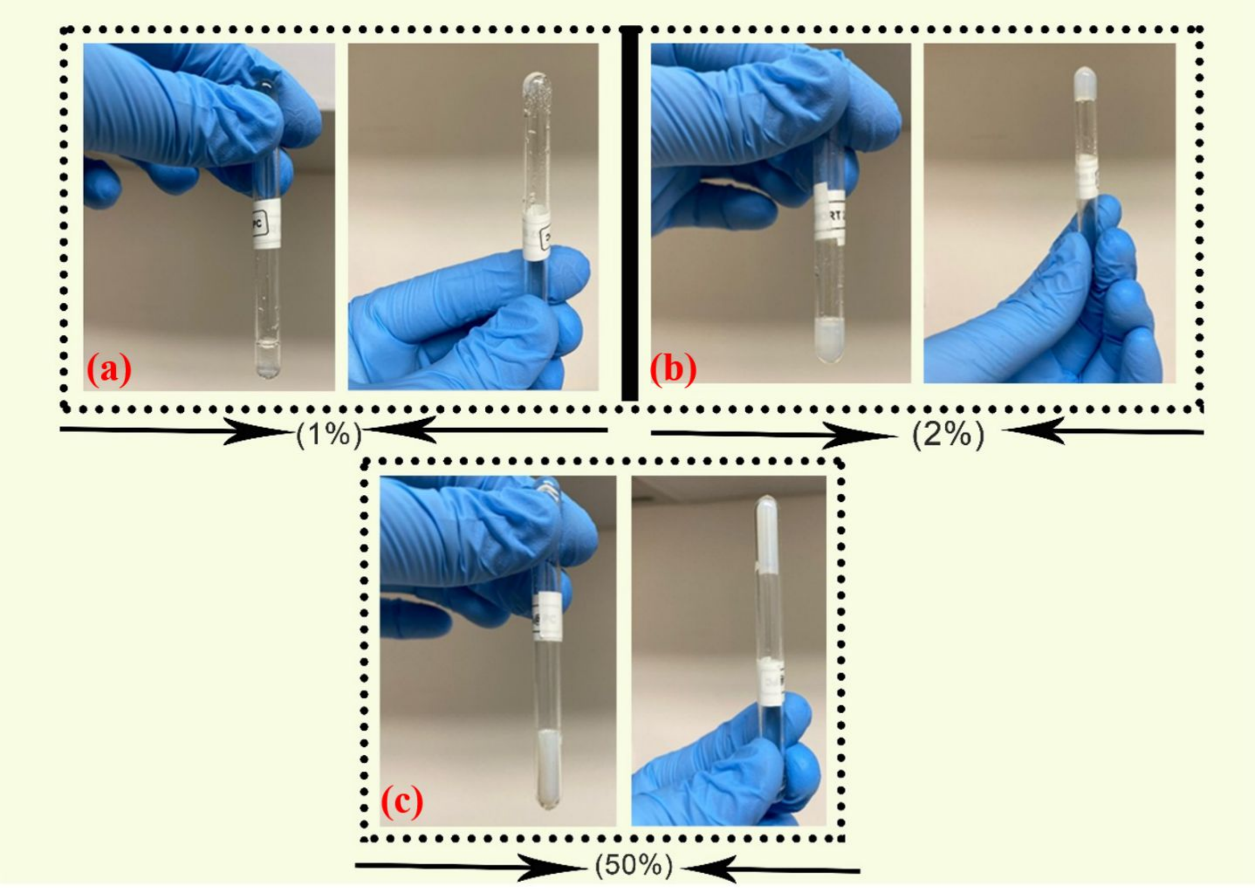

Fig.S5 (a) 1% G2 in PC (systems below MGC) (**b)** 2% G2 in PC (systems at MGC) **(c)** 50% G2 in PC (systems above MGC)
